# Statement of Retraction: Knockdown of zinc finger protein 267 suppresses diffuse large B-cell lymphoma progression, metastasis, and cancer stem cell properties

**DOI:** 10.1080/21655979.2024.2299579

**Published:** 2024-02-20

We, the Editors and Publisher of the journal *Bioengineered*, have retracted the following article:

Hua Yang, Linmei Wang, Yingbin Zheng, Guiming Hu, Hongyan Ma & Liyun Shen (2022) Knockdown of zinc finger protein 267 suppresses diffuse large B-cell lymphoma progression, metastasis, and cancer stem cell properties, Bioengineered, 13:1, 1686-1701, DOI: 10.1080/21655979.2021.2014644

Since publication, significant concerns have been raised about the integrity of the data and reported results in the article. When approached for an explanation, the authors did not provide their original data or any necessary supporting information. As verifying the validity of published work is core to the integrity of the scholarly record, we are therefore retracting the article. All authors listed in this publication have been informed.

We have been informed in our decision-making by our policy on publishing ethics and integrity and the COPE guidelines on retractions.

The retracted article will remain online to maintain the scholarly record, but it will be digitally watermarked on each page as ‘Retracted’.

